# Effects of Dinoprost-Tromethamine and Collection Method on Semen Quality of Commercial Boars

**DOI:** 10.3390/vetsci13080791

**Published:** 2026-08-08

**Authors:** Luciano Bianco do Amaral, Eder Batalha Araujo, Pedro Nacib Jorge-Neto, Dominike Prediger Delazeri, Janine de Camargo, Mariana Groke Marques, Ricardo Zanella

**Affiliations:** 1 School of Agricultural Sciences, Innovation and Business, Graduate Program in Bioexperimentação, University of Passo Fundo, Passo Fundo 99052-900, RS, Brazil; 107145@upf.br (L.B.d.A.); janinedecamargo@upf.br (J.d.C.); 2 Topigs Norsvin, Guarapuava 80420-210, PR, Brazil; eder.batalha@topigsnorsvin.com.br; 3 IMV Technologies, Campinas 13030-300, SP, Brazil; pepovet@gmail.com; 4 School of Agricultural Sciences, Innovation and Business, Veterinary Medicine Program, University of Passo Fundo, Passo Fundo 99052-900, RS, Brazil; 190266@upf.br; 5 Embrapa Suínos e Aves, Concórdia 89715-899, SC, Brazil; mariana.marques@embrapa.br

**Keywords:** Dinoprost, semen collection, boars, motility, spermatozoa

## Abstract

Semen quality is a critical factor for the success of artificial insemination programs in swine production, and is influenced by collection method, genetic lineage, collection interval and pharmacological interventions. This study evaluated the effects of Dinoprost-tromethamine (DP; Lutalyse^®^, Zoetis, Parsippany, NJ, USA), a prostaglandin F_2_α analog, on boar semen quality in a commercial stud, considering manual versus semi-automatic collection systems, four genetic lines and resting time between collections. A total of 653 boars (146 Large White, 210 Landrace, 282 Duroc and 15 Hybrid [Duroc × Pietrain]) were monitored over 12 months, resulting in 22,977 semen collections. DP was administered intramuscularly as a single dose of 1 mL per boar (6.71 mg of active compound; approximately 0.028 mg/kg body weight), 20 min before semen collection, exclusively to animals classified with low libido (scores 1–3), whereas boars with normal libido (scores 4–6) remained untreated. Semen parameters (ejaculate volume, sperm concentration, total motility and kinematic traits assessed by CASA) were analyzed using mixed-effects models including collection method, DP treatment, genetic line and their interactions. Semi-automatic collection increased ejaculate volume and total motility but resulted in lower sperm concentration compared with manual collection. DP administration increased sperm motility and sperm concentration while reducing ejaculate volume, with the greatest improvement in motility observed in Hybrid boars and more modest or nonsignificant responses in the other genetic lines. Longer collection intervals were associated with higher sperm concentration, whereas ejaculate volume and motility were not affected by resting time. These findings indicate that the impact of DP, collection method and collection interval on semen quality is breed-dependent and highlight the need to tailor reproductive management protocols according to boar genetics, libido and collection technology.

## Simple Summary

Efficient pork production relies heavily on successful artificial insemination, but semen quality often varies due to differences in male pig genetics and handling methods. This study aimed to evaluate how semi-automatic versus manual collection systems, four pig breeds (Large White, Landrace, Duroc, and Hybrid), and the administration of a reproductive hormone Dinoprost-tromethamine (DP) influence semen traits. By evaluating over twenty-two thousand collection procedures, we found that the semi-automatic system generally increased ejaculate volume and sperm movement across most breeds, though manual collection yielded more concentrated samples in Large White pigs. Furthermore, the hormone treatment successfully boosted sperm movement and concentration, showing its strongest impact on Hybrid pigs, while extending resting days between collections slightly increased sperm concentration. We conclude that a single standardized management strategy cannot be applied identically to all boars. These findings are highly valuable to society because they offer commercial breeding studs practical guidelines to customize their routines based on specific animal breeds and technologies. This tailored approach enhances reproductive efficiency, reduces production waste, and supports a more sustainable and cost-effective food supply chain.

## 1. Introduction

The success of assisted reproduction programs in animals depends directly on semen quality and the efficiency of artificial insemination procedures [1]. Semen parameters such as ejaculate volume, sperm concentration, motility, and viability are key indicators of male reproductive performance [2,3]. Several factors influence these characteristics, including boar management, environmental conditions, nutrition, and the use of pharmacological substances that may enhance or decrease ejaculate quality. Among these substances, prostaglandins have attracted interest because of their physiological effects on the male reproductive system [4,5,6].

Dinoprost-tromethamine, a synthetic analog of prostaglandin F_2_α (PGF_2_α), is widely used in reproductive management, primarily for estrous cycle control in females [7,8]. The action of prostaglandins is mediated through specific receptors coupled to G-proteins that activate the cAMP cascade and stimulate calcium release via the phosphatidylinositol pathway [9]. These receptors are located on cell membranes and are classified according to the prostaglandin type they bind. Although the mechanisms by which prostaglandins influence male sexual behavior are not fully understood, in pigs PGF_2_α appears to stimulate regions of the central nervous system, particularly the hypothalamus, a key regulator of reproductive function [10]. PGF_2_α also acts on smooth muscle contractility, including the epididymis and seminal vesicles [5]. Administration of DP before semen collection may facilitate sperm transport and the release of seminal plasma components, potentially improving ejaculate quality [11,12].

In addition to its contractile effects on the reproductive tract, DP may influence hormonal release, such as testosterone, by stimulating oxytocin secretion and enhancing Leydig cell activity. This hormonal modulation can affect spermatogenesis and the final quality of semen. Studies in rats, cattle, pigs, and sheep have demonstrated variable responses to prostaglandin use, emphasizing the need for species- and context-specific evaluation [13]. Despite evidence suggesting potential benefits of DP during semen collection, results remain inconsistent [5,10]. We hypothesized that administering DP to boars with reduced libido would improve ejaculate volume, sperm concentration and motility, and that these effects would depend on genetic lineage and semen collection method. Specifically, we expected that semi-automatic collection and Hybrid boars would show the greatest improvements in semen quality, whereas responses in other breeds and collection systems would be more variable. Therefore, this study aimed to evaluate the effects of DP administration on semen quality in boars subjected to manual and semi-automatic collection methods, with emphasis on its practical applicability in boar stud management.

## 2. Materials and Methods

### 2.1. Animals and Study Design

This study was approved by the Animal Ethics Committee (CEUA) under protocol number 005/2023 and was conducted during routine operations of a commercial boar semen collection and processing center located in Paraná, Brazil, throughout 2024. Semen from 653 boars (Sus scrofa) representing four commercial genetic lines was evaluated: Large White (n = 146), Landrace (n = 210), Duroc (n = 282), and Hybrid (Duroc × Pietrain cross; n = 15).

The study followed a retrospective longitudinal design where 653 boars were monitored over a 12-month period, accounting for the total of 22,977 semen collections analyzed (Table 1). Semen was collected using two systems: a manual gloved-hand technique and a semi-automatic collection system based on an artificial cervix attached to a semi-automated dummy (BoarMatic^®^, Minitüb GmbH, Tiefenbach, Deutschland). Of the 22,977 semen collections analyzed, 18,208 were performed using the semi-automatic system and 2475 using manual collection, while 454 and 1840 collections involved automatic and manual collection with DP administration, respectively, as detailed in Table 1. This distribution reflects the routine management of the stud, where most ejaculates are obtained using the semi-automatic system, and allows evaluation of potential method-related effects on semen quality. The study factors included collection method (manual or semi-automatic), administration or non-administration of Dinoprost-tromethamine, a synthetic analog of prostaglandin F_2_α (Lutalyse^®^, Zoetis, Parsippany, NJ, USA), boar genetic line, and collection interval (resting time between collections).

### 2.2. Semen Collection Procedures

The double-gloved hand technique was used to manually grasp the glans penis during semen collection. In semi-automatic semen collection systems, the penis-fixation structure used a device that minimizes exposure of the ejaculate to ambient air during collection. Although an artificial cervix is employed, a manual handling step is still required until the artificial cervix is secured to the dummy. In both methods, the collection facility comprised a pre-collection pen, where cleaning and emptying of the preputial diverticulum were performed. The collection pen (1.0 × 2.5 m) was equipped with a semi-automated dummy (BoarMatic^®^, Minitüb GmbH, Tiefenbach, Deutschland) that was used for both type of semen collections. An adjacent technician pit (0.9 m in height) was positioned alongside the collection pen to facilitate operator access during semen collection [14].

### 2.3. Libido Scoring and DP Administration

Boars selected for treatment were classified by libido scores ranging from 1 to 6, based on mounting behavior and time to ejaculate. The scoring system was defined as follows: score 1, failure to mount within 40 min; score 2, mounting after >30 min; score 3, mounting between 20 and 30 min; score 4, mounting between 10 and 20 min; score 5, mounting within 5–10 min with normal ejaculatory behavior; and score 6, mounting within <5 min with normal ejaculatory behavior. Dinoprost-tromethamine (DP; Lutalyse^®^, Zoetis, Parsippany, NJ, USA), a synthetic analog of prostaglandin F_2_α (PGF_2_α), was administered intramuscularly only to boars classified with low libido (scores 1–3). The dose used in the study was 1 mL per boar, corresponding to 6.71 mg of active compound (approximately 0.028 mg/kg of body weight, considering an average boar weight of 240 ± SD kg). DP was administered as a single intramuscular injection approximately 20 min before each semen collection session. Boars with normal libido (scores 4–6) did not receive DP and served as controls. DP administration followed the routine management protocol of the commercial stud and was applied exclusively to boars classified as having low libido. No experimental allocation or randomization was performed. Consequently, comparisons between DP-treated and untreated boars should be interpreted as observational associations rather than causal treatment effects.

The study followed a retrospective observational design based on routine semen collection records obtained from a commercial boar stud over a 12-month period. Ejaculate volume, sperm concentration, and total motility were analyzed using a mixed-effects model including collection method, genetic line, and libido class as fixed effects, boar as a random effect, and DP treatment applied only within the low-libido group. Libido score was included as a fixed effect in the statistical model to account for potential baseline differences between treated and untreated boars. Sperm motility was assessed by computer-assisted semen analysis (CASA; IVOS II, Hamilton Thorne, Beverly, MA, USA).

### 2.4. Semen Evaluation and CASA

Collection intervals (resting time between successive collections) ranged from 3 to 7 days and were recorded for each boar. The effect of resting time was evaluated using regression analysis to determine its association with ejaculate volume, sperm concentration and motility. Semen analysis included measurement of ejaculate volume, sperm concentration (million/mL), and total motility (%). In addition, sperm kinematic parameters were assessed using computer-assisted semen analysis (CASA; IVOS II, Hamilton Thorne, USA), including curvilinear velocity (VCL), straight-line velocity (VSL), average path velocity (VAP), linearity (LIN), straightness (STR) and amplitude of lateral head displacement (ALH). These parameters were used to characterize sperm movement patterns and to complement the evaluation of total motility.

### 2.5. Statistical Analyses

Homogeneity of variances was tested using Levene’s test, and residual normality was evaluated with the Anderson–Darling test. Statistical analyses were conducted in RStudio v. 4.5.2. Fixed effects included collection method, DP administration, genetic line, and their interactions. Resting time, repetitions, and residual error were treated as random effects. When significant (*p* < 0.05), means were compared using Tukey’s test. Regression analysis was applied to assess the effect of collection interval on ejaculate volume, sperm concentration, and motility. Comparisons were performed both between breeds and within breeds, contrasting boars treated with DP versus untreated animals, as well as manual versus semi-automatic collection methods. This approach allowed us to evaluate main effects and interactions of genetic line, collection system and DP administration on semen parameters.

## 3. Results

### 3.1. Main Effects of Collection Method and DP Treatment

A total of 22,977 semen collections were evaluated, distributed across four collection systems and four genetic lines (Table 1). The highest number of collections was performed using the automatic collection system (n = 18,208), whereas Landrace (n = 9599) and Duroc (n = 8955) represented the most frequent boar breeds utilized in the study.

**Figure 1:**
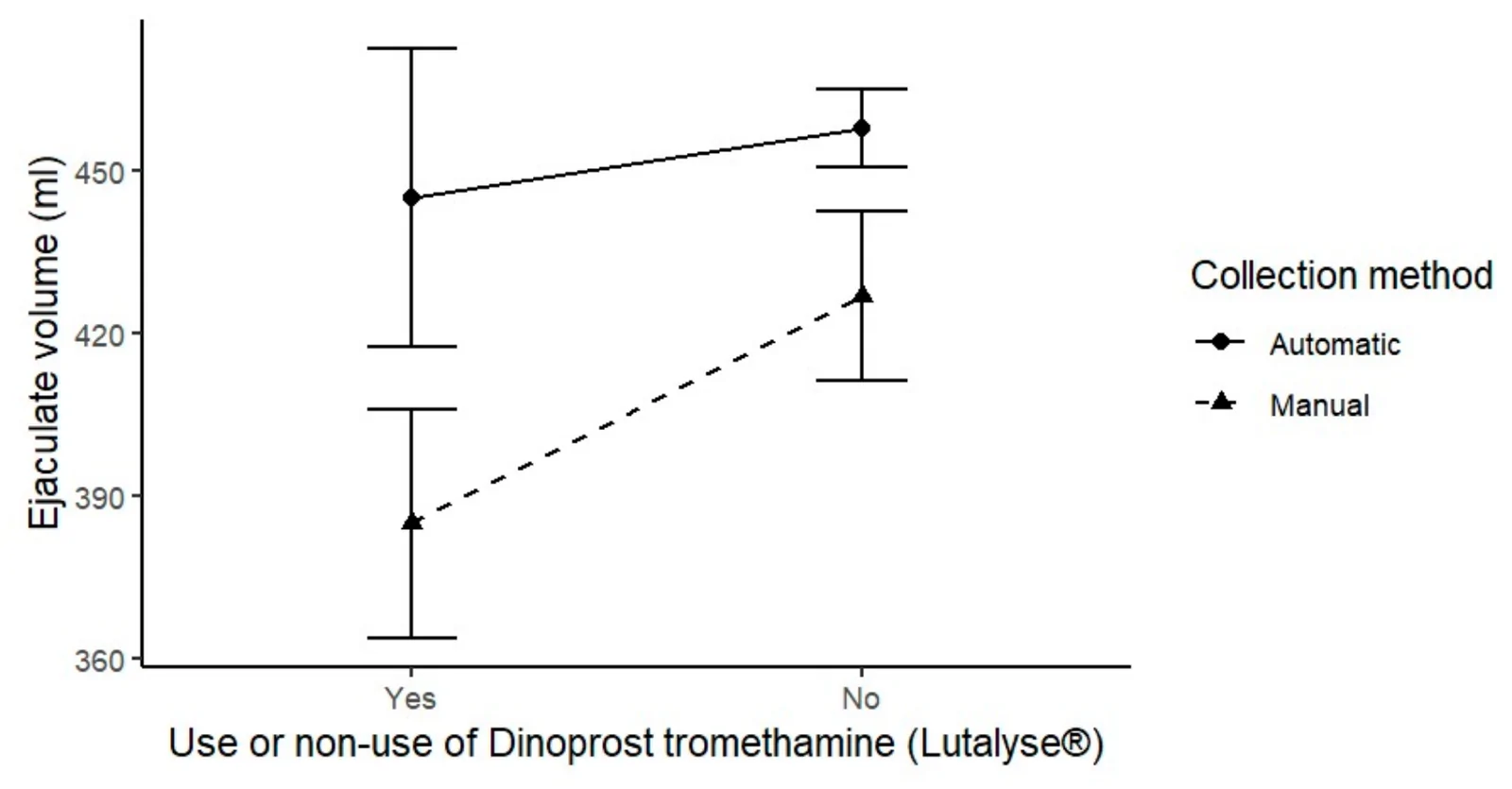
Ejaculate volume according to collection method and use or non-use of PGF_2_α (Dinoprost-tromethamine Lutalyse^®^).

A significant interaction between collection method and Dinoprost-tromethamine (Lutalyse^®^) administration was observed for ejaculate volume and sperm motility (*p* < 0.05). The ejaculate volume was higher when semen was collected using the semi-automatic system when compared with the manual collection regardless of Lutalyse^®^ administration (*p* < 0.05) Figure 1.

**Figure 2:**
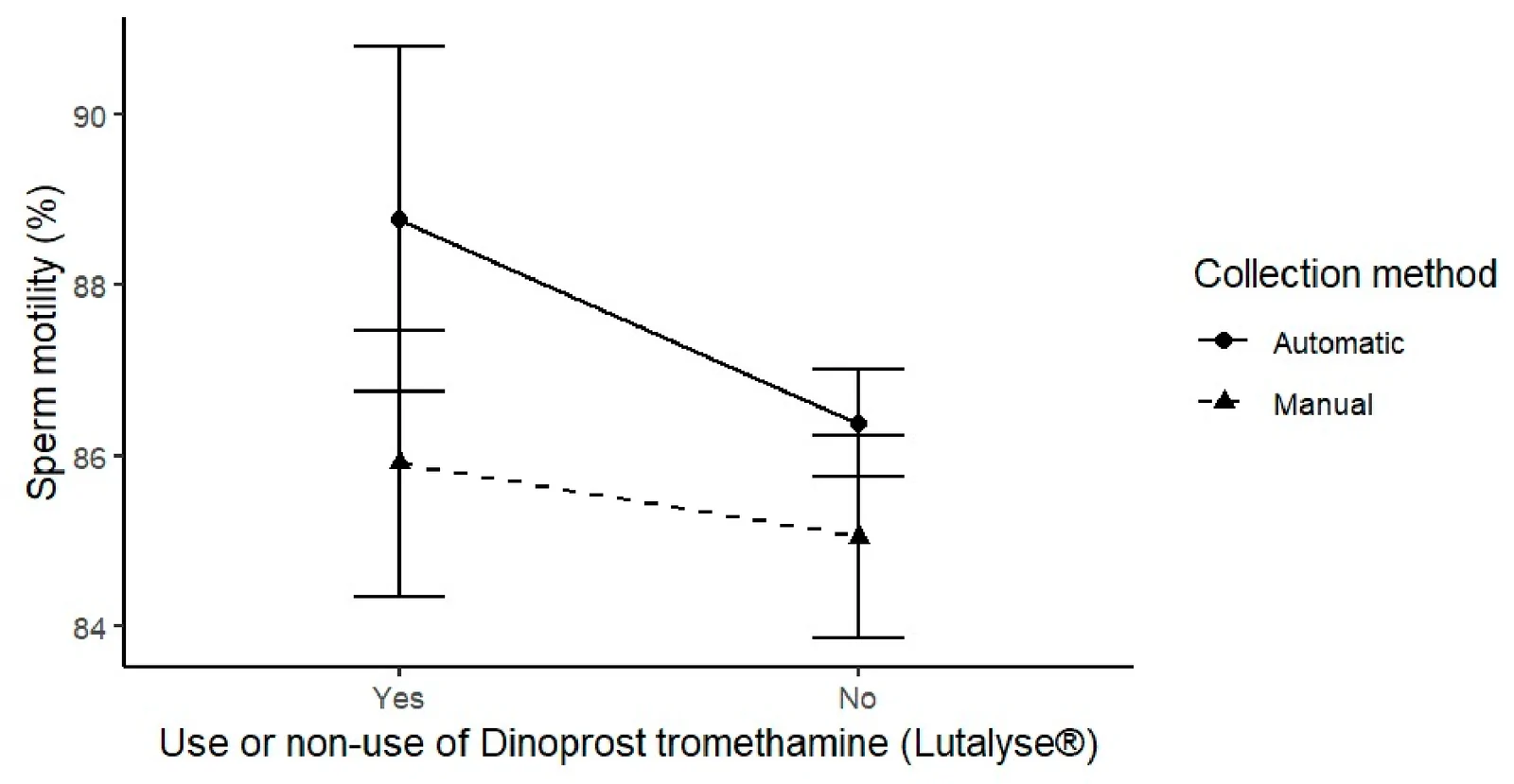
Sperm motility according to collection method and use or non-use of PGF_2_α (Dinoprost-tromethamine Lutalyse^®^). DP was administered exclusively to boars with low libido (Yes) (scores 1–3), whereas high-libido boars (scores 4–6) served as untreated control.

Higher sperm motility was observed when the semen was collected using the semi-automatic system when compared with the manual collection. Furthermore, DP administration to low-libido boars (scores 1–3) increased the percentage of motile sperm within both collection methods when compared with untreated boars (scores 4–6) (*p* < 0.05) Figure 2.

### 3.2. Interaction Between Collection Method and Genetic Line

**Figure 3:**
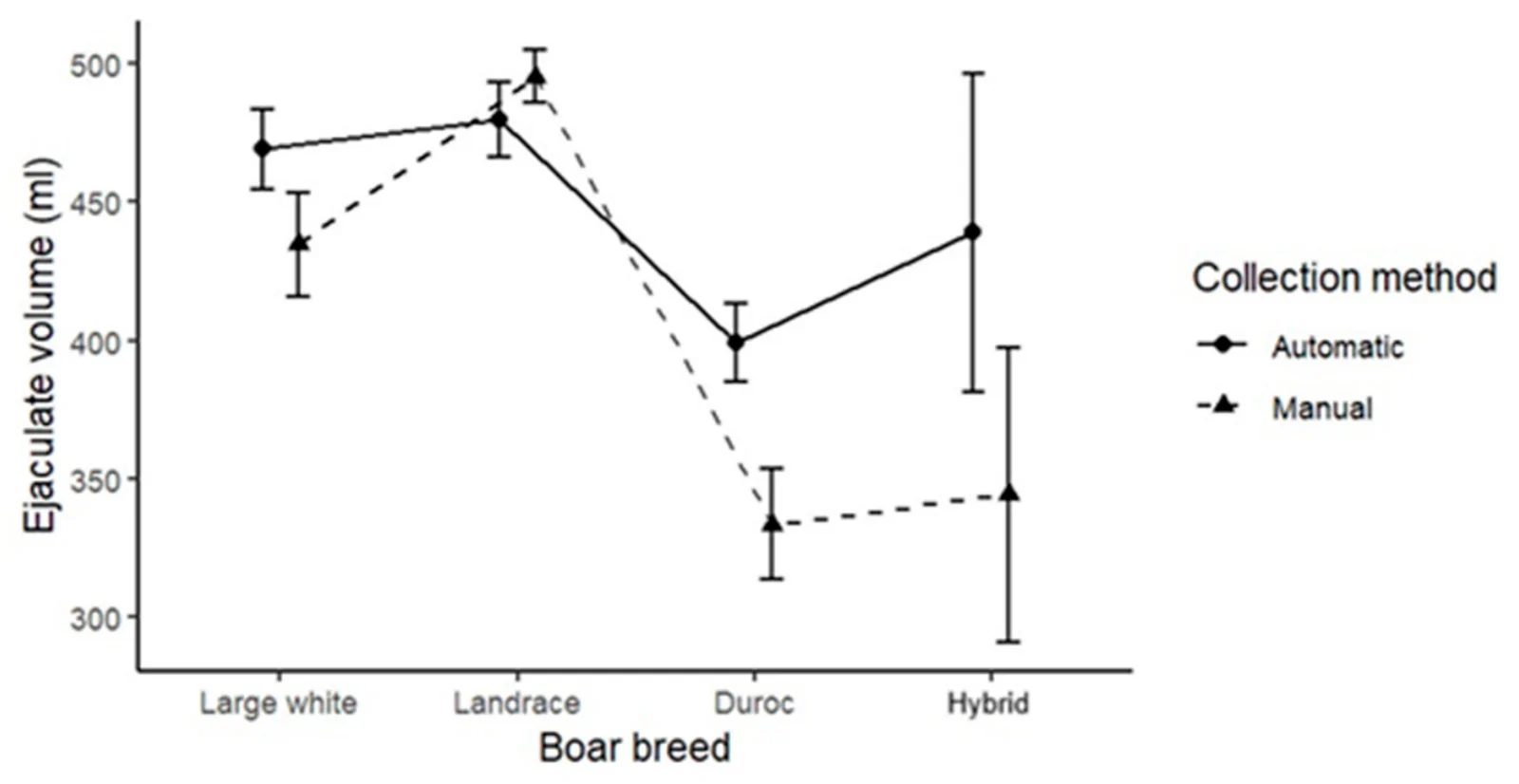
Ejaculate volume according to collection method and boar breed.

**Figure 4:**
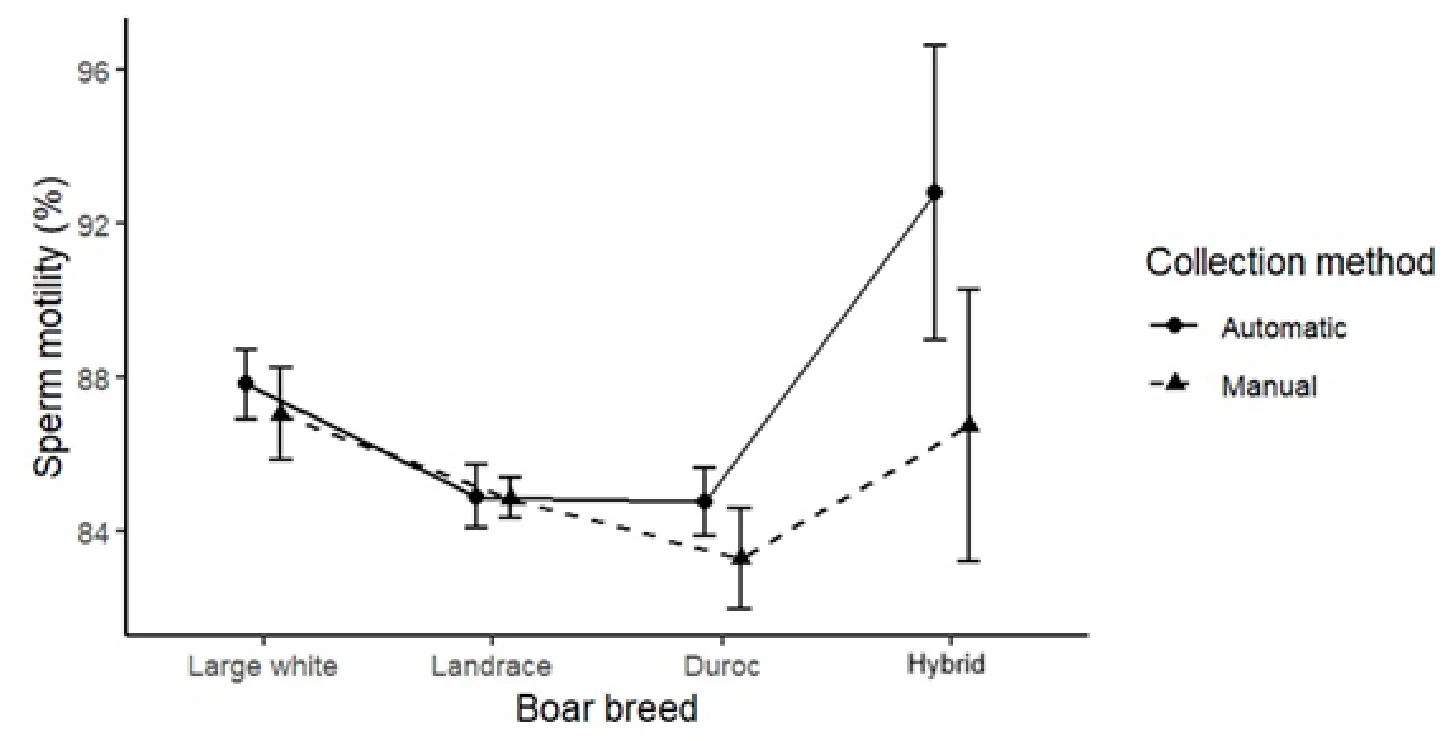
Sperm motility according to collection method and boar breed.

An interaction between collection method and genetic line was observed for ejaculate volume, motility, and sperm concentration (*p* < 0.05; Table 2), indicating that both genetic and management factors jointly influence semen quality. Large White, Duroc, and Hybrid boars produced greater ejaculate volumes when semen was collected automatically than manually, whereas Duroc boars showed higher ejaculate volume during manual collection in specific combinations of method and DP use (*p* < 0.05; Figure 3). Sperm concentration was higher in Large White boars collected manually (*p* < 0.05), while for the other genetic lines, sperm concentration did not differ between collection methods (*p* > 0.05). Regarding motility, Hybrid and Duroc boars presented higher values when semen was collected automatically (*p* < 0.05; Figure 4). Although Hybrid boars showed the greatest increase in sperm motility, the limited number of Hybrid animals receiving DP reduces statistical precision and prevents broad extrapolation of these breed-specific findings.

Sperm concentration was higher in Large White boars collected manually (*p* < 0.05; Figure 4). For the other genetic lines, sperm concentration did not differ between methods (*p* > 0.05). Regarding motility, Hybrid and Duroc boars presented higher values when semen was collected automatically (*p* < 0.05; Figure 4).

### 3.3. Interaction Between Genetic Line and DP Administration

**Figure 5:**
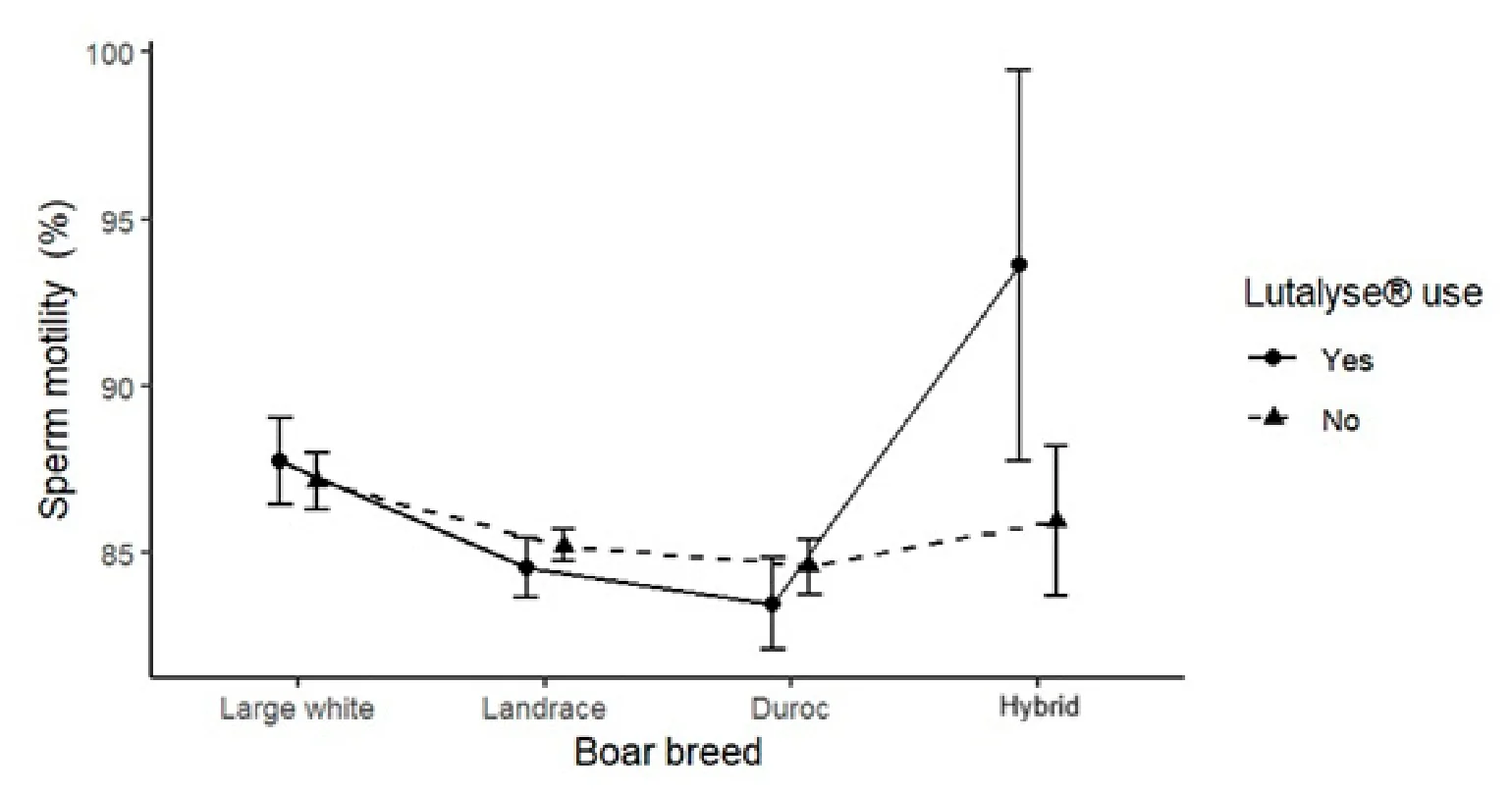
Sperm motility according to PGF_2_α (Lutalyse^®^) use and boar breed.

The interaction between genetic line and DP administration was significant for sperm motility (*p* < 0.05). Motility was higher in Hybrid boars treated with Lutalyse^®^ (*p* < 0.05; Figure 5). For the other breeds, DP did not affect motility (*p* > 0.05). Although Hybrid boars exhibited the largest motility improvement following DP treatment, the number of Hybrid animals receiving DP was limited, which restricts the generalization of breed-specific conclusions and should be interpreted with caution.

Overall, semi-automated collection yielded higher ejaculate volume and motility, but lower sperm concentration compared to manual collection (*p* < 0.05). DP use increased sperm concentration and motility while reducing ejaculate volume (*p* < 0.05; Table 2). Breed-specific effects included higher ejaculate volume for Landrace boars and higher sperm concentration and motility for Large White and Hybrid boars (*p* < 0.05), as shown in Table 3. Sperm concentration increased by 3.49 million/mL per additional resting day (*p* < 0.05; R2 = 4%). Neither volume nor motility showed significant regression with resting time (*p* > 0.05).

## 4. Discussion

This study demonstrates that semen collection method, genetic line, and Dinoprost-tromethamine administration significantly influence boar semen quality. In summary, semi-automatic collection was associated with greater ejaculate volume and higher motility but lower sperm concentration than manual collection, Dinoprost-tromethamine administered to low-libido boars increased sperm concentration and motility while reducing volume, Landrace boars produced larger ejaculates, and Large White and Hybrid boars showed higher sperm concentration and motility. Previous reports have indicated that automated or standardized collection procedures provide greater control of environmental and physiological conditions, reducing stress and improving ejaculate quality. Lower manual handling and shorter exposure to external factors may preserve sperm integrity by reducing bacterial contamination [14,15,16].

This study showed that the interaction between collection method and genetic line affected ejaculate volume, motility, and sperm concentration, indicating that both genetic and management factors jointly influence semen quality. Large White, Duroc, and Hybrid boars produced greater ejaculate volumes when collected automatically, likely due to better adaptation of these genotypes to standardized, low-stress conditions [16,17,18]. Conversely, in our study Duroc boars showed higher ejaculate volume during manual collection suggesting that they may respond more positively to individualized physical stimuli, possibly linked to behavioral or anatomical differences affecting ejaculatory reflexes [19,20,21]. These breed-dependent patterns are consistent with previous reports showing that Landrace and Large White boars generally produce higher ejaculate volumes than terminal breeds such as Duroc, whereas crossbred boars often exhibit intermediate or superior total sperm outputs due to combined effects of volume and concentration [22,23].

Sperm concentration was higher in Large White boars collected manually, indicating that individualized handling may favor emission of more concentrated ejaculates. For the other genetic lines, sperm concentration did not differ between methods suggesting breed-specific sensitivity to collection conditions [17]. Regarding motility, Hybrid and Duroc boars presented higher values when semen was collected automatically. This supports the hypothesis that standardized, low-stress collection environments enhance sperm functional integrity [24,25]. Taken together, these interactions between method and breed indicate that the choice between manual and semi-automatic collection should not be uniform across all sires but rather tailored to the genetic line and the desired balance between ejaculate volume, sperm concentration and motility in commercial studs [4,19].

DP, a PGF_2_α analog, increases contractions of accessory glands and ejaculatory ducts, promoting release of seminal fluid with improved nutritional and physiological support for spermatozoa [26,27,28]. This study showed that the interaction between genetic line and DP administration was significant for sperm motility. For the other breeds, DP did not affect motility (*p* > 0.05), confirming breed-dependent responses [17,18]. Our observation that Dinoprost-tromethamine increased sperm concentration while reducing ejaculate volume in low-libido boars is partially consistent with recent large-scale observational data from commercial AI centers, where PGF_2_α-treated boars exhibited higher sperm concentration but lower ejaculate volume across multiple breeds, although overall motility tended to be reduced in treated animals [29]. These discrepancies likely reflect differences in treatment protocols, target populations and baseline fertility, and highlight that PGF_2_α effects should be interpreted within the specific management context of low-libido animals in commercial studs [10,20,27,29].

Regarding the main effects observed in this study, it is noteworthy that overall, semi-automated collection yielded higher ejaculate volume and motility, but lower sperm concentration compared to manual collection. In parallel, the main effects of pharmacological intervention showed that DP use increased sperm concentration and motility while reducing ejaculate volume. Furthermore, looking at individual genetics, breed-specific effects included higher ejaculate volume for Landrace boars and higher sperm concentration and motility for Large White and Hybrid boars. With respect to collection interval, sperm concentration increased by 3.49 million/mL per additional resting day, whereas ejaculate volume and motility showed comparatively smaller changes, indicating that frequency of collection predominantly influences the number of sperm available per ejaculate rather than the fluid fraction or movement quality [30,31,32]. When analyzing the impact of management intervals, our findings indicated that sperm concentration increased by 3.49 million/mL per additional resting day, although high variability indicated influence of other physiological and management factors. Finally, it is important to highlight that neither volume nor motility showed significant regression with resting time, consistent with previous reports indicating that frequent collections may impair epididymal function and sperm maturation [30].

A limitation of this study is that Dinoprost-tromethamine was administered only to boars classified as having lower libido scores. Therefore, the observed treatment effects should be interpreted with caution, as treated and untreated animals may differ in their baseline reproductive characteristics. In addition, the study followed a retrospective observational design in a commercial boar stud, where collection method and treatment decisions were made according to routine operational criteria rather than randomized allocation. As in other commercial AI center studies, this non-randomized context introduces potential selection bias and confounding by factors such as boar temperament, technician, facility conditions and season, which may influence semen quality independently of the evaluated interventions [20,29]. Consequently, our results should be viewed as ecologically valid associations that reflect real-world management conditions, but they do not allow definitive causal conclusions about the effects of collection method, Dinoprost-tromethamine or collection interval; future controlled and randomized trials are needed to confirm these relationships and refine evidence-based recommendations [10,27,29].

This study demonstrates that semen collection method, genetic line, and Dinoprost-tromethamine administration significantly influence boar semen quality. Collectively, these findings emphasize the importance of tailoring reproductive management according to boar breed, collection system, and pharmacological interventions to optimize semen quality and reproductive efficiency in commercial studs.

## 5. Conclusions

This study shows that boar semen quality depends on collection method, genetic line and targeted use of Dinoprost-tromethamine in low-libido sires, with semi-automatic collection increasing ejaculate volume and motility and manual collection favoring higher sperm concentration in specific breeds such as Large White, indicating that collection strategies should be adapted to breed and production goals. In commercial studs, management protocols should therefore combine breed-specific choice of collection system with selective DP administration to low-libido boars and appropriate resting intervals, aiming to optimize semen volume, concentration and motility rather than applying a single standardized approach to all animals. Because DP was administered only to low-libido boars and the study followed an observational design without randomization, the observed treatment effects represent associations rather than definitive causal relationships, and future controlled trials are warranted to confirm these findings and refine evidence-based guidelines for boar reproductive management.

## Figures and Tables

**Table 1:** Number of semen collections by collection system and genetic line (automatic collection, automatic with PGF_2_α, manual with PGF_2_α, manual collection).

Groups	Boar Breed				Total per Group
	Large White	Landrace	Duroc	Hybrid	
Automatic collection	3706	5913	8461	128	18,208
Automatic with PGF_2_α	143	156	153	2	454
Manual	197	2056	189	33	2475
Manual with PGF_2_α	198	1474	152	16	1840
Total per boar breed	4244	9599	8955	179	22,977

**Table 2:** Analysis of sperm parameters in boars, considering the collection method (manual vs. semi-automatic), genetic breed, and the use of PGF_2_α (Dinoprost-tromethamine-DP).

Parameter	Collection Method (M)		PGF_2_α (L)		SEM	Probability				
	Automatic	Manual	Yes	No		M	L	M × L	M × Breed	L × Breed
Volume (mL)	447.0	402.0	411.0	438.0	8.3	<0.01	<0.01	<0.01	<0.01	0.24
Concentration (Million/mL)	199.0	203.0	205.0	197.0	7.9	<0.01	<0.01	0.25	<0.01	0.82
Motility (%)	87.6	85.5	87.3	85.7	0.5	0.05	0.02	0.01	0.02	0.02

SEM = standard error of the mean; M = collection method; L = PGF_2_α treatment; M × L = interaction between collection method and PGF_2_α treatment; M × Breed = interaction between collection method and breed; L × Breed = interaction between PGF_2_α treatment and breed; Tukey’s test, *p* < 0.05.

**Table 3:** Effects of breed on boar semen quality.

Parameter	Swine Breed				SEM1	*p*
	Large White	Landrace	Duroc	Hybrid		
Volume (mL)	452.0 b	487.0 a	367.0 c	392.0 c	9.6	<0.01
Concentration (Million/mL)	212.0 a	177.0 c	192.0 b	222.0 a	8.5	<0.01
Motility (%)	87.4 a	84.9 b	84.0 b	89.8 a	0.6	<0.01

SEM = standard error of the mean. Different letters within the same row indicate significant differences among breeds by Tukey’s test (*p* < 0.05).

## Data Availability

The raw data supporting the conclusions of this article will be made available by the authors on request.

## Abbreviations

The following abbreviations are used in this manuscript:

CASA	Computer-Assisted Semen Analysis
CEUA	Animal Ethics Committee (Comissão de Ética no Uso de Animais)
DP	Dinoprost-Tromethamine
PGF_2_α	Prostaglandin F_2_α
SEM	Standard Error of the Mean
